# Clinical Features of HIV Arthropathy in Children: A Case Series and Literature Review

**DOI:** 10.3389/fimmu.2021.677984

**Published:** 2021-07-20

**Authors:** Michael J. Harrison, Nicola Brice, Christiaan Scott

**Affiliations:** ^1^ Fort Beaufort Provincial Hospital, Amathole District, Eastern Cape, South Africa; ^2^ Division of Paediatric Rheumatology, Department of Paediatrics and Child Health, Red Cross War Memorial Children’s Hospital, Cape Town, South Africa; ^3^ University of Cape Town, Rondebosch, Cape Town, South Africa

**Keywords:** paediatric HIV, inflammatory arthritis, Africa, autoimmunity, non-communicable disease, musculoskeletal manifestations

## Abstract

**Background:**

HIV infection has been associated with a non-erosive inflammatory arthritis in children, although few published reports exist. This study describes the clinical, laboratory and imaging features of this noncommunicable disease in a series of HIV-infected children in South Africa.

**Methods:**

A database search was conducted to identify HIV-infected children enrolled in a Paediatric Rheumatology service in Cape Town, South Africa between 1 January 2010 and 31 December 2020. Retrospective data were collected from individuals classified with HIV arthropathy, based on a predefined checklist. Demographic, clinical, laboratory, sonographic, therapeutic, and outcomes data were extracted by chart review. Descriptive statistical analysis was performed using R (v4.0.3).

**Results:**

Eleven cases of HIV arthropathy were included in the analysis. Cases predominantly presented in older boys with low CD4+ counts. Median age at arthritis onset was 10.3 years (IQR 6.9 – 11.6) and the male-female ratio was 3.0. The median absolute CD4+ count was 389 cells/uL (IQR 322 – 449). The clinical presentation was variable, with both oligoarthritis and polyarthritis being common. Elevated acute phase reactants were the most consistent laboratory feature, with a median ESR of 126 mL/h (IQR 67 – 136) and median CRP of 36 mg/L (IQR 25 – 68). Ultrasonography demonstrated joint effusions and synovial hypertrophy. Response to therapy was slower than has generally been described in adults, with almost all cases requiring more than one immunosuppressive agent. Five children were discharged in established remission after discontinuing immunotherapy, however outcomes data were incomplete for the remaining six cases.

**Conclusions:**

In this case series, HIV arthropathy was associated with advanced immunosuppression. Therapeutic modalities included immunomodulators and antiretroviral therapy, which consistently induced disease remission although data were limited by a high rate of attrition. Prospective studies are needed to define and understand this HIV-associated noncommunicable disease.

## Introduction

Autoimmune disease has been reported in the context of human immunodeficiency virus (HIV) infection since the origin of the HIV pandemic. The spectrum of HIV-associated rheumatic disease has evolved over time, likely reflecting the effects of antiretroviral therapy (ART) on survival and immunological function (1–4). Variable prevalence rates (11 - 72%) have been reported for musculoskeletal disorders in HIV-infected adults (5–8). However, data are scarce for the paediatric population.

South Africa is positioned at the epicentre of the global HIV pandemic, with a prevalence of 19% in adults and approximately 340,000 HIV-infected children (9, 10). The number of new paediatric HIV infections decreased almost five-fold from 47,000 in 2010 to 10,000 in 2020, reflecting reduced vertical transmission. However, a considerable treatment gap remains in the paediatric population, with only 63% of HIV-infected children receiving ART and only 46% of those on ART achieving viral suppression (11). Despite this, mortality in HIV-infected children has declined dramatically (12–14). As life expectancy in this high-risk group increases, a greater number of children may present with rheumatic sequelae of HIV infection and therapy.

HIV infection disrupts the immunological milieu, giving rise to a broad spectrum of inflammatory and immune-mediated syndromes (1–3). In the setting of advanced HIV infection, multiple immune aberrations contribute to loss of self-tolerance. Proposed mechanisms include the destruction of CD4+ T-lymphocyte populations, autoreactive CD8+ T-lymphocyte responses, T-regulatory cell dysfunction, exposure of autoantigens, B-lymphocyte activation, generation of autoantibodies and immune complexes, molecular mimicry, and direct viral invasion of immunologically active tissue (1, 15–20). A combination of these mechanisms have been implicated in the diverse immune-mediated non-communicable sequelae of HIV infection. Conversely, the balance of immune activation and deficiency induced by HIV infection appears to be protective against certain autoimmune phenomena, including rheumatic heart disease and systemic lupus erythematosus (SLE) (2, 21, 22).

Inflammatory arthritis appears to be associated with HIV infection, although the existing literature is limited by conflicting definitions of disease, inconsistent application of classification systems, and variable availability of relevant diagnostic tests and expertise (2). The spondyloarthropathies have been extensively investigated in relation to HIV infection (23–26). These conditions are strongly associated with the human leucocyte antigen (HLA)-B27 allele. This HLA haplotype is extremely uncommon in Black African populations (27, 28), and resultantly the spondyloarthropathies were rarely reported in Africa prior to the HIV pandemic. The prevalence of these conditions in sub-Saharan Africa appears to have increased dramatically in tandem with the HIV pandemic (29–31). HIV-related immunosuppression can reduce disease activity and even induce remission in patients with established rheumatoid arthritis (RA) (32). Early reports describing this phenomenon led to the conviction that HIV and RA were mutually exclusive disorders. However, this position has since shifted in the face of a growing body of evidence indicating that HIV-positive individuals can develop an entity clinically indistinguishable from RA (8, 33–35), with a reported prevalence of 0.1 - 5.0% (31, 36–38). Most cases present in immunocompetent HIV-infected individuals (34, 39).

HIV arthropathy has been defined as a self-limiting, asymmetrical, nonerosive, oligo- or polyarthritis that predominantly involves the lower limbs and typically resolves within six weeks (1, 2, 40). Some authors have suggested that rheumatoid factor (RF) and HLA-B27 negativity be required for its diagnosis (41, 42), in order to differentiate the entity from RA or spondyloarthropathies. Reveille and colleagues define adult-onset HIV arthropathy as “arthritis of the large joints, lasting less than six weeks, in the absence of either HLA B27 positivity, or radiological changes … distinct from any other recognized rheumatological entity, with no discerned infective triggers” (41). A prevalence of 0.4 – 13.8% has been reported in adults (2, 37, 43–47), with higher rates reported in studies conducted in predominantly African populations (31, 44, 48). HIV arthropathy is generally described in the setting of advanced HIV (8, 49). When performed, synovial fluid analysis reveals sterile inflammation, with nonspecific features of chronic synovitis on biopsy (49, 50). Tubulo-reticular inclusions, HIV-associated p24 antigen, and HIV DNA-containing dendritic cells have been identified in the synovial tissue of affected individuals, suggesting a direct viral aetiology (40, 49, 51, 52). It has been proposed that HIV arthropathy may represent a type of reactive arthritis triggered by the virus itself (2, 53), however the precise pathophysiology has yet to be described.

The optimal management of HIV arthropathy has not been completely defined. In the current understanding of the disease, therapy is primarily directed at the underlying cause. Therefore, effective ART and immune reconstitution are the cornerstones of management. Specific therapy may also be directed against the inflammatory component of HIV arthropathy. Chloroquine is frequently used and has been shown to have beneficial anti-viral activity in addition to its anti-inflammatory effects (54–56). Nonsteroidal anti-inflammatories are routinely used for symptomatic control. Systemic corticosteroids may be used, although the duration of therapy should be limited to reduce toxicity. Similarly, intra-articular corticosteroids are considered safe and efficacious. The role of other immunomodulator therapies, including methotrexate, sulfasalazine, and biologic agents, remains undefined.

There are few published reports on HIV arthropathy in children. A case series from Durban, South Africa described 35 HIV-infected children with arthritis (50). This series reported a male predominance, and a mean age of onset of 5.5 years. The most common pattern of articular involvement was asymmetrical polyarthritis, and RF and HLA-B27 were negative in almost all cases. Another report from the same centre described seven HIV-infected children with both arthritis and uveitis, in whom no cause other than HIV could be found (57). Two isolated case reports of paediatric HIV-associated arthritis have been published in India (58, 59). Arthritis was the presenting feature of HIV infection in both cases. A case series of musculoskeletal manifestations in HIV-infected children in India reported 3 cases of inflammatory arthritis, all of which occurred in older girls with advanced immunosuppression (60). Another report from India described a single case of HIV arthropathy, and three cases of HLA-B27-associated arthritis in HIV-infected children (61). A study of juvenile idiopathic arthritis from Zambia reported 7 cases of arthritis in HIV-infected children, but did not provide details of subgroup analysis (62). A large South African study which screened perinatally HIV-infected adolescents and HIV-uninfected controls for musculoskeletal disorders reported a low prevalence of musculoskeletal abnormalities, which were associated with advanced immunosuppression and longer duration of ART (63). A pre-ART study conducted among 40 HIV-infected children in the United States found no clinically significant rheumatological manifestations, although high rates of hypergammaglobulinemia, autoantibody positivity, and the presence of circulating immune complexes were reported (64). A Mexican study of 26 HIV-infected children described several cases of Raynaud’s phenomenon and peripheral vasculitis, and one child with septic arthritis (65). Although no cases of inflammatory arthritis were observed in the North American children, arthralgia was commonly reported in both studies (64, 65).

There is very limited published data on paediatric HIV arthropathy, both globally and in sub-Saharan Africa. This study aimed to describe features of HIV arthropathy in a case series of children who presented to a Paediatric Rheumatology centre in Cape Town, South Africa.

## Materials and Methods

### Aims, Design, and Setting

This study was conducted at two large referral centres in Cape Town, South Africa. The study was approved by the Human Research Ethics Committee of the University of Cape Town (HREC 011/2021). The aim of this study was to describe HIV arthropathy in terms of clinical presentation, laboratory characteristics, sonographic features, therapy, and outcomes in a case series of HIV-infected children in South Africa. Relevant data were collected by retrospective review of medical records.

### Participants

All HIV-infected paediatric patients enrolled in the Paediatric Rheumatology clinics at either Red Cross War Memorial Children’s Hospital or Groote Schuur Hospital between 1 January 2010 and 31 December 2020 were retrospectively identified by searching the digital databases of the two centres, using the keywords *human immunodeficiency disease/HIV* and *retroviral disease/RVD.* Chart review was undertaken to identify those patients classified as having HIV arthropathy, using a predefined checklist adapted from Reveille and colleagues’ definition (41). HIV-infected children with other rheumatic disorders were excluded from the analysis.

### Data Collection

Demographic, clinical, and laboratory data were extracted by review of participant details, case notes and laboratory results. Clinical manifestations, laboratory results, and medical therapies were captured at initial presentation and subsequent follow up. Radiographic and ultrasonographic data were extracted by review of saved images and reports for existing studies. Therapeutic modalities were analysed as binary variables, with positivity conferred by a record of an agent having been previously prescribed. Response to therapy was measured by extrapolating clinical data recorded at outpatient follow up consultations, such as the number of active involved joints, and linear scales of patient/parent-rated functional limitation and clinician-rated functional limitation, relative to dates of starting or stopping therapies, or changing doses.

### Statistical Analysis

Characterisation of demographics and clinical presentation was summarised using descriptive statistics. Demographic, clinical and laboratory variables were expressed as medians with interquartile ranges, as the frequency distributions for these data were skewed. Treatment and outcomes data were summarized using line graphs, in which duration of a specific therapy was plotted against a measure of disease activity. Data were analysed through statistical package R version 4.0.3.

## Results

An initial search identified 22 HIV-infected patients, from a total of 900 children enrolled in the Paediatric Rheumatology clinics. Eleven patients were excluded, due to alternative diagnoses (sarcoidosis, n=1; systemic sclerosis, n=1; uveitis, n=3; Osgood-Schlatter, n=2; vasculopathy, n=1; pernio, n=1; cellulitis, n=1) or incomplete data (n=1). A total of 11 cases of paediatric-onset HIV arthropathy were included in the analysis.

### Clinical Presentation

Most HIV arthropathy cases presented in older children, with a median age at arthritis onset of 10.3 years (IQR 6.9 – 11.6). There was a significant male predominance (male-female ratio 3.0). Although the median age at HIV diagnosis was 7.6 years (IQR 5.6 – 10.6), it was considered likely that most of these patients acquired HIV infection *via* vertical transmission. Most cases presented in the setting of advanced HIV infection, with a median absolute CD4+ count of 389 cells/uL (IQR 322 – 449) and median CD4+ proportion of 19.5% (IQR 14.8 – 25.0) at presentation, although there was one outlier with an absolute CD4+ count of 2364 cells/uL (CD4+ proportion 49.0%). Arthritis was the presenting feature of HIV infection in 4 cases. Six children (55%) were ART naïve at the time of presentation with arthritis. The remaining five children had all defaulted ART for a period of more than six months prior to presentation.

**Figure 1:**
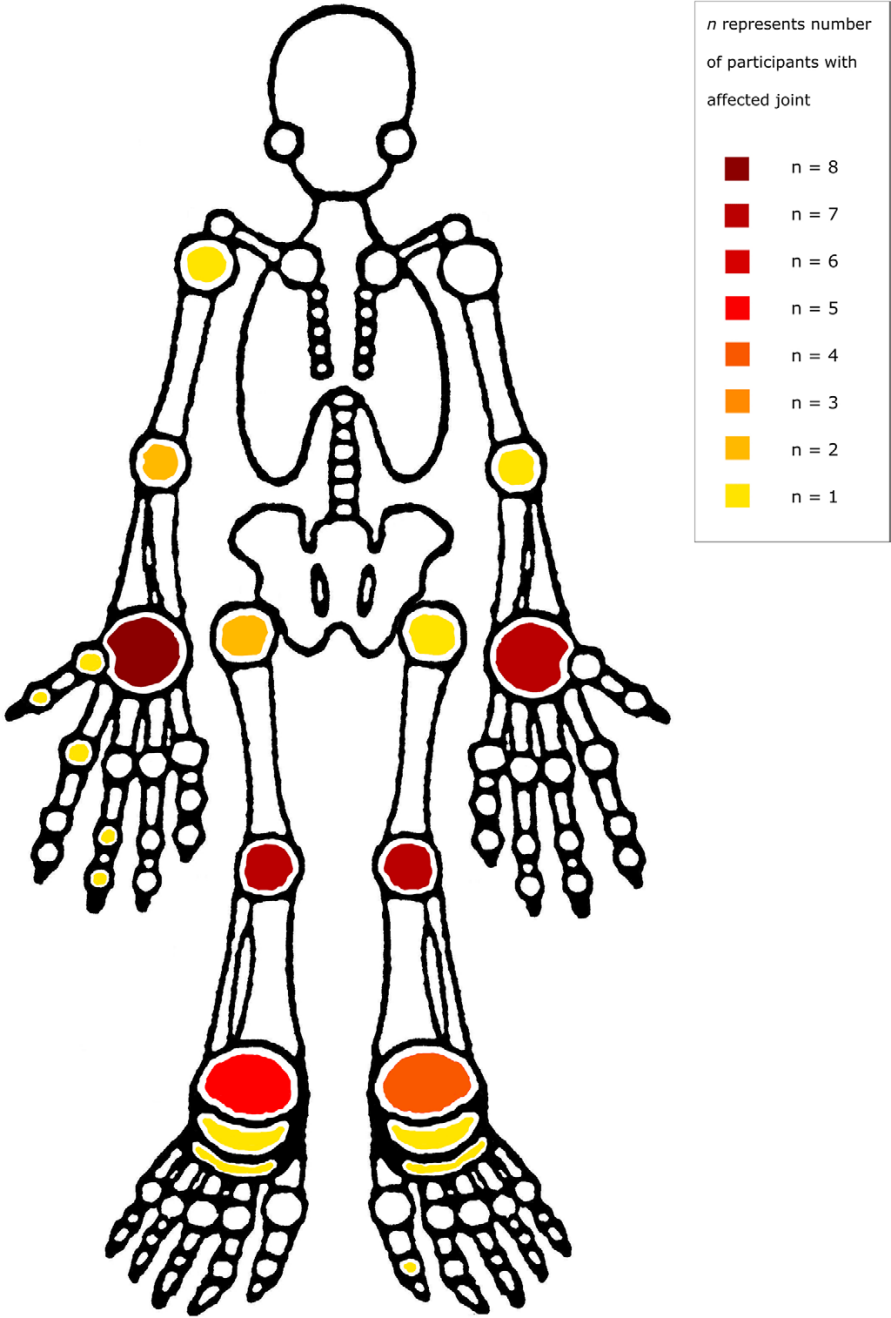
Distribution of joint involvement.

Clinical features are summarized in **Table 1**. The pattern of articular involvement was variable, with six cases of oligoarthritis and five cases of polyarthritis. The median number of involved joints was 4 (IQR 3- 6). The distribution of joint involvement is shown in **Figure 1**. All eleven children had large joint involvement (wrists, n=8; knees, n=7; ankles, n=5; hips, n=2; elbows, n=2; shoulders, n=1), which was asymmetrical in nine cases. In addition, four children had asymmetrical small joint involvement (finger interphalangeal, n=3; toe interphalangeal, n=1; metacarpophalangeal, n=2; midtarsal, n=1) and one child had symmetrical involvement of the subtalar and midtarsal joints. Enthesitis was found in four patients, and one child had dactylitis. Associated clinical features included lymphadenopathy (n=5), hepatomegaly (n=4), splenomegaly (n=2), and parotidomegaly (n=2). Seven children had associated skin rashes, which were classified as papular pruritic eruption (n=4), psoriasis (n=1), erythema nodosum (n=1), and scabies (n=1). Two children had uveitis. The nutritional status of these patients was generally poor, with 89% being classified as underweight-for-age and all being stunted. Approximately half (54.5%) had a personal history of previous tuberculosis (TB), of which four cases were pulmonary and one case was intra-abdominal. There was also one case of TB monoarthritis, involving the left knee, in a child with pre-existing HIV arthropathy involving both wrists and the interphalangeal joint of the left great toe. Other opportunistic infections included oral candidiasis, mucocutaneous herpes simplex ulcers, varicella zoster meningitis, parvovirus B19 aplastic anaemia, and cytomegalovirus gastroenteritis.

**Table 1:** Clinical presentation.

	CD4 count at HIV diagnosis, cells/uL (%)	Pattern of articular involvement (number of active joints)	Associated rheumatic features	Associated clinical features	Opportunistic infections	Comorbidities	Nutritional status
1	431 (20)	Polyarthritis (6)	Erythema nodosum	LAD, hepatomegaly	Pulmonary TB	–	Mild UWFA
							Mild stunting
2	752 (29)	Oligoarthritis (3)	Tenosynovitis	Splenomegaly, PPE	–	–	Normal WFA
							Mild stunting
3	444 (15)	Oligoarthritis (4)	Enthesitis	PPE	Oral candidiasis	–	Moderate UWFA
							Mild stunting
4	453 (15)	Oligoarthritis (2)	–	LAD, hepatomegaly, parotidomegaly, PPE	–	–	Moderate UWFA
							Mild stunting
5	161 (11)	Oligoarthritis (4)	–	LAD, HSM	Abdominal TB, parvovirus B19 aplastic anaemia	Severe acute malnutrition	Severe UWFA
							Moderate stunting
6	375 (19.5)	Polyarthritis (10)	Enthesitis, myalgia	Oral ulcers, clubbing, scabies	Pulmonary TB	Post-TB bronchiectasis	*
7	237 (18)	Oligoarthritis (3)	Dactylitis, myalgia	LAD, PPE	TB monoarthritis, CMV enteritis	–	Severe UWFA
							Severe stunting
8	389 (21)	Oligoarthritis (2)	Enthesitis, uveitis	Bilateral cataracts, hepatomegaly	Pulmonary TB, VZV meningitis	–	Severe UWFA
							Severe stunting
9	2364 (49)	Polyarthritis (6)	–	LAD	Chronic HSV ulcers	Severe acute malnutrition	Severe UWFA
							Severe stunting
10	282 (14)	Polyarthritis (8)	Enthesitis	–	–	Obstructive hydrocephalus	Severe UWFA
							Severe stunting
11	361 (34)	Polyarthritis (5)	Psoriasis	Parotidomegaly	Pulmonary TB	–	*

Rt, right; Lt, left; LAD, lymphadenopathy; UWFA, underweight-for-age; WFA, weight-for-age; MCPJ, metacarpophalangeal joint; IPJ, interphalangeal joint; PIPJ, proximal interphalangeal joint; DIPJ, distal interphalangeal joint; TB, tuberculosis; HSV, herpes simplex virus; CMV, cytomegalovirus; VZV, varicella zoster virus.

*Insufficient data.

### Laboratory and Imaging Characteristics

Laboratory features are summarised in **Table 2**. Synovial aspirate was performed in 3/11 cases. The typical findings were of a lymphocyte-predominant, exudative effusion with negative bacterial and mycobacterial analyses. Synovial biopsy was performed in 4/11 cases. Histological analyses demonstrated nonspecific subacute and chronic synovitis. All microbiological analyses were negative (Ziehl-Neelson, Auramine, Gram, Brown-Brenn, and periodic acid-Schiff stains; CMV immunohistochemistry; bacterial and mycobacterial cultures), except for a repeat synovial biopsy sample which cultured Micrococcus species, which was interpreted as a contaminant. Autoimmune serological data are presented in **Table 3**.

**Table 2:** Laboratory features.

	n/N (%)
Leucocytosis (WCC > 11.0 x10^9^ cells/L)	2/11 (18.2)
Leucoopenia (WCC <4.0 x10^9^ cells/L)	1/11 (9.1)
Neutropenia (ANC < 1.5 x10^9^ cells/L)	2/10 (20.0)
Lymphopenia (ALC < 1.4 x10^9^ cells/L)	3/9 (33.3)
Anaemia (Hb < 11.0 g/dL)	6/11 (54.5)
Thrombocytosis (Platelet count > 400 x10^9^ cells/L)	5/11 (45.5)
CRP > 10 mg/L	11/11 (100)
ESR > 30 mL/h	10/11 (90.9)
	Median (IQR)
WCC, x10^9^ cells/L	8.8 (8.0 – 9.2)
ANC, x10^9^ cells/L	4.2 (3.4 – 5.6)
ALC, x10^9^ cells/L	1.9 (1.3 – 2.1)
Hb, g/dL	10.7 (9.9 – 11.9)
Platelet count, x10^9^ cells/L	361 (335 – 503)
CRP, mg/L	36 (25 – 68)
ESR, mL/h	126 (67 – 136)

WCC, white cell count; ANC, absolute neutrophil count; ALC, absolute lymphocyte count; Hb, haemoglobin; CRP, C-reactive protein; ESR, erythrocyte sedimentation rate; n, number; N, sample; IQR, interquartile range.

**Table 3:** Autoantibodies.

	n tested (%)	n positive
ANA	6/11 (54.5)	0
Anti-dsDNA	3/11 (27.3)	0
RF	6/11 (54.5)	0
HLA-B27	3/11 (27.3)	1
ASO	3/11 (27.3)	0
Anti-DNase B	3/11 (27.3)	0

ANA, antinuclear antibodies; anti-dsDNA, anti-double stranded deoxyribonucleic acid antibodies; RF, rheumatoid factor; HLA-B27, human leukocyte antigen B27; ASO, antistreptolysin O; anti-DNase B, anti-deoxyribonuclease B.

Joint ultrasound data were available for 7/11 cases (63.6%); findings included joint effusions (6/7) and synovial hypertrophy (4/7). Joint plain film radiographic data were available for 3/11 cases (27.3%); two X-rays were normal, and one showed a joint effusion.

### Treatment and Outcome

**Figure 2:**
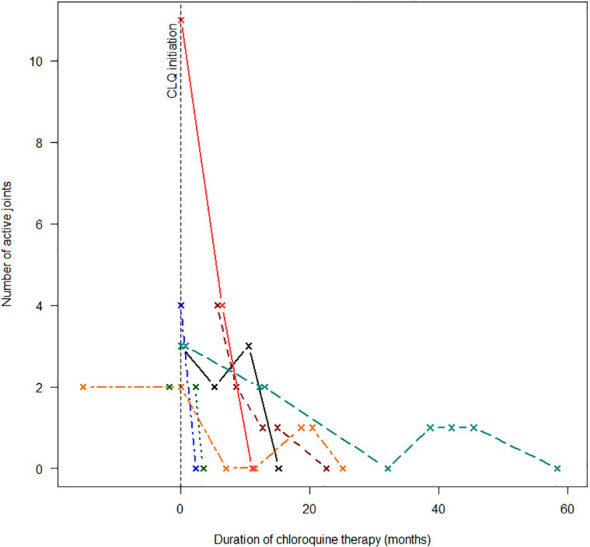
Response to chloroquine therapy.

Immune-directed therapeutic modalities are summarized in **Table 4**. Non-steroidal anti-inflammatory drugs (NSAIDs) were used in all cases. Oral steroids were used in seven children. Intra-articular steroids were used in three of the remaining four children, and the fourth child never returned for follow-up after initial presentation. Two children required intravenous pulse steroid therapy to control severe disease flares. Clinical responses to chloroquine, methotrexate, and prednisone therapy are illustrated in **Figures 2** **–** **4**. Chloroquine was either withheld or stopped in four children, due to possible or confirmed TB infection, and methotrexate was stopped in one child with active TB infection.

**Table 4:** Therapeutic modalities.

	n (%)	Duration at remission, in months Median (IQR)	Total duration, in months Median (IQR)
NSAID	11 (100.0)	–	–
CLQ	9 (81.8)	12.0 (9.1 – 18.9)	22.8 (18.0 – 48.1)
MTX	5 (45.5)	15.0 (11.5 – 20.0)	27.7 (11.7 – 30.1)
Leflunomide	1 (9.1)	–	–
Oral prednisone	7 (63.6)	13.8 (12.4 – 18.7)	22.0 (18.0 – 32.9)
Intravenous steroid therapy	2 (18.2)	–	–
Intraarticular steroid therapy	4 (36.4)	–	–

NSAID, nonsteroidal anti-inflammatory drug; CLQ, chloroquine; MTX, methotrexate; n, number; IQR, interquartile range.

**Figure 3:**
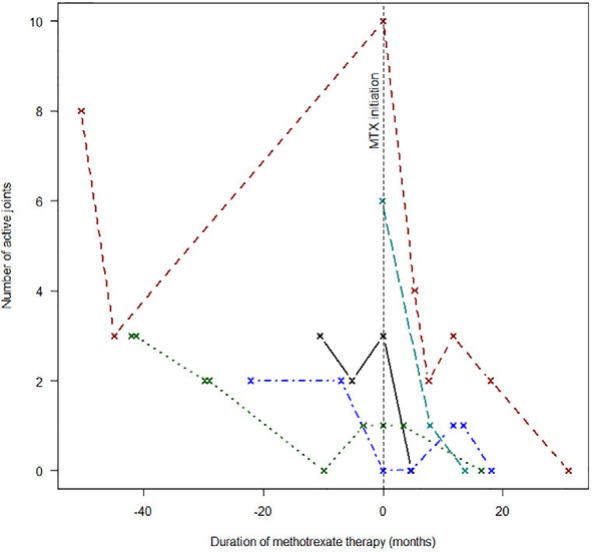
Response to methotrexate therapy.

**Figure 4:**
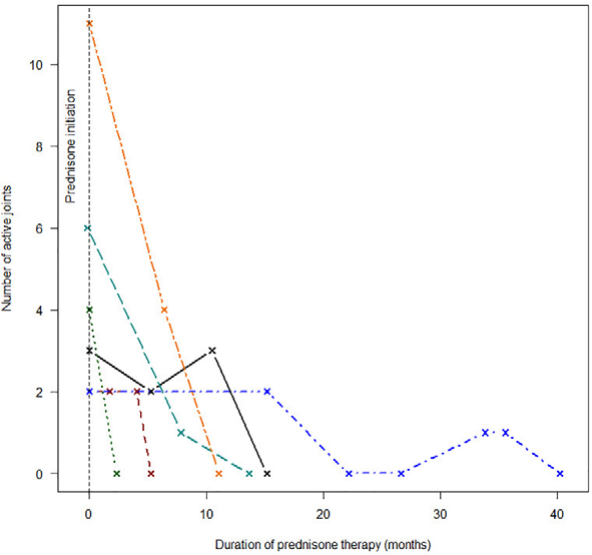
Response to prednisone therapy.

Five children were discharged in established remission after discontinuing immunotherapy. Two children were lost to follow-up immediately after presentation. Another four children were lost to follow-up after longer periods of routine assessment, however all four were in remission at last assessment.

ART was initiated in all eleven children, at a median age of 7.4 years (IQR 6.3 – 10.1). Viral suppression (defined as a single recorded viral load <100 copies/uL) was achieved in 9/11 children (82%), with a median time to viral suppression of 45.5 months (IQR 9.3 – 124.9). Over the study period, the mean change in absolute CD4+ count was -57.1 cells/uL and the mean change in CD4+ proportion was +3.0%. Five children were changed to a second-line ART regimen during the study period.

## Discussion

A plethora of research efforts have attempted to explore and address the diverse biopsychosocial challenges which shape the lives of children living with HIV/AIDS. However, musculoskeletal manifestations of HIV infection have not been prioritized and remain poorly defined. Rheumatological disorders in HIV-infected children appear to be relatively uncommon, and paediatric practice has been shaped by data generated in adult populations.

While the number of children with advanced HIV/AIDS has declined substantively in the current era of universal ART, significant treatment gaps continue to affect children living with HIV. A recent study integrating paediatric HIV data sources in South Africa reported that approximately one quarter of HIV-infected children remain undiagnosed, compared to a corresponding estimate of one tenth of adults (66). ART coverage in children was estimated to be only 51.2%. This data suggests that the dramatic reductions in AIDS-related under-five mortality have largely been driven by preventive strategies, such as prevention of mother-to-child transmission, rather than effective management of existing paediatric HIV cases. In this context, the effective recognition and management of both communicable and non-communicable sequelae of paediatric AIDS remains imperative. This report adds to the small body of literature on paediatric HIV arthropathy, which has emerged as a clinical entity distinct from other rheumatic disorders of childhood. As in adults, paediatric HIV arthropathy occurs in the setting of advanced immunosuppression (8, 49). Additionally, it appears to predominantly affect older children (50, 60). In this case series, the median age of arthritis onset was 10.3 years (IQR 6.9 – 11.6), which is similar to existing literature. Most children had advanced immunosuppression at the time of presentation, with a median CD4+ proportion of 19.5% (IQR 14.8 – 25.0).

A high male-female ratio was evident in this case series. This finding is consistent with reports of a male predominance in both children and adults with HIV arthropathy (23, 50, 67) and contrasts with reports of older children with advanced HIV disease without arthropathy (68–70). The mechanisms contributing to the observed sex difference remain unclear. Biological sex has a profound effect on the immune dysregulation that characterises HIV infection. Numerous studies in children and adults have demonstrated lower levels of viraemia in females (71–74). Women are over-represented amongst HIV-infected individuals with the capacity to spontaneously suppress viraemia without ART, known as elite controllers. Across the literature, approximately 80% of adult elite controllers and 90% of paediatric elite controllers are female (71, 75–78). Sex differences in other non-communicable HIV-associated comorbidities, such as cardiovascular disease, neurocognitive disorders and malignancies, have been linked to substantial differences in immune activation and regulation between males and females living with HIV (79–81).

In comparison with adults, most untreated HIV-infected children follow a rapid course of disease progression. At one year of life, 35–50% of untreated, perinatally infected infants will have progressed to AIDS or died (71, 82–85), although it should be noted that outcomes vary widely across different settings, with environmental exposures playing a significant role in early life. By comparison, the median survival time in untreated adults is approximately 10 years (86). It is therefore surprising that a significant proportion of children (5-10%) exhibit the immunological phenomenon of non-progression, which is extremely uncommon in adults (75, 87). Non-progressors are ART-naïve HIV-infected individuals who do not experience immunosuppression but maintain normal CD4+ counts despite ongoing viraemia (75, 88). Most of the children in this series experienced long delays to ART initiation, with median age at ART initiation being 7.4 years (IQR 6.3 – 10.1). This suggests that delay to ART initiation and subsequent HIV progression may be a risk factor for HIV arthropathy in children. Conversely, at least some of these children may have been transient non-progressors, facilitating their survival into later childhood. HLA haplotype-encoding genes influence the rate of disease progression in HIV infection (88–91), with certain HLA haplotypes being recognized as protective. However, several such alleles are also implicated in the pathogenesis of specific rheumatic syndromes. In a large case-control study based in Zambia, López-Larrea and colleagues demonstrated that the presence of the HLA-B*5703 allele was independently associated with both HIV non-progression and the development of spondyloarthropathy (92). Other reports have demonstrated high frequencies of HLA haplotypes associated with spontaneous HIV control in individuals with psoriasis and spondyloarthropathy (93, 94). An intriguing possibility in the pathogenesis of HIV arthropathy is the notion that an as-of-yet unknown factor may be partially protective against HIV progression and confer an increased risk of inflammatory arthritis.

A variable pattern of articular involvement was observed in this case series, with six children presenting with oligoarthritis and five having polyarthritis. This differs from reports of adult-onset HIV arthropathy, in which asymmetrical oligoarthritis predominates, although symmetrical polyarthritis and monoarthritis have also been described (23, 95). Polyarthritis was the commonest presentation in the largest existing report of arthritis in HIV-infected children (50). Most of the children in this case series (9/11) had symmetrical involvement of the large joints, particularly wrists and knees. Existing literature has reported preferential involvement of the lower limbs, particularly knees and ankles, in both adults and children (23, 49, 50). Enthesitis (3/11) was less prevalent than reported elsewhere (50, 96), and only one case was associated with psoriasis. Other observed clinical features (such as papular pruritic eruption, stunting, lymphadenopathy, hepatosplenomegaly and parotidomegaly) likely reflect the advanced stage of HIV infection in which arthropathy presents, rather than a true association. Opportunistic infections were common in this case series, particularly TB (6/11) including two extrapulmonary cases.

**Figure 5:**
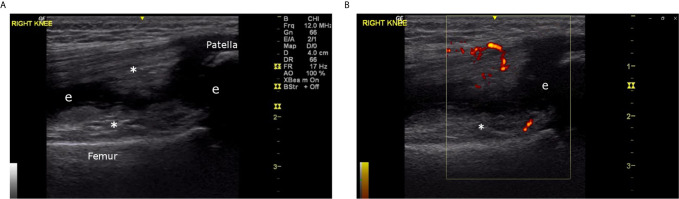
**(A)** Right knee, superior longitudinal view, greyscale (6 year old male, Case 2), demonstrating abundant proliferative synovitis (*) and effusion (e). **(B)** Right knee, superior longitudinal view, color Doppler (6 year old male, Case 2), demonstrating proliferative synovitis with intense 3+ Doppler signal (D).

The most consistent laboratory finding was elevated acute phase reactants, which were present in more than 90% of cases. ESR was usually elevated to a greater extent than CRP. The median ESR was 126 mL/h (IQR 67 – 136), compared to a median CRP of 36 mg/L (IQR 25 – 68). Joint ultrasound data were available for seven cases (63.6%) in this series. Prominent sonographic features included joint effusion (6/7) and synovial hypertrophy (4/7). Synovial hypertrophy was distinctive; all four cases were described as having abundant or exuberant hypertrophy, relative to findings in patients with juvenile idiopathic arthritis (**Figures 5** **–** **7**). Published evidence on the sonographic features of HIV arthropathy is scarce for adults and children alike. However, several reports have highlighted the radiographic features of joint effusions, juxta-articular osteopenia, joint space narrowing, and marginal erosions in adults with HIV-associated arthritides (95, 97, 98). Findings on plain radiograph are generally similar to those seen in patients with RA, with the exception of periosteal reaction and proliferative bone formation, which are more specific to HIV arthropathy (97, 99).

Approximately half of the children in this case series (45.5%) were discharged in established remission after discontinuing immunotherapy. The remaining six children were lost to follow up; four of them were in remission at last assessment. Two children had no follow up data, having been lost to follow up immediately after initial presentation. Notably, all cases with reliable follow-up data had joint disease persisting beyond 6 weeks, which contrasts with Reveille and colleagues’ classic definition of adult-onset HIV arthropathy as a self-limiting condition (41). This phenomenon has been observed in some adults, who develop a chronic arthropathy associated with functional impairments (100).

**Figure 6:**
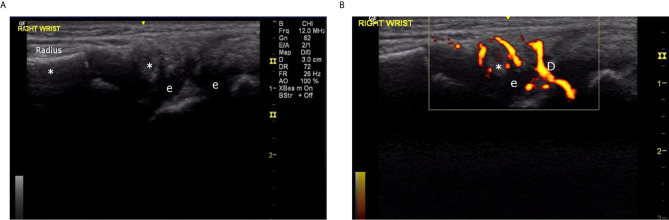
**(A)** Right wrist, 4^th^ compartment, longitudinal view, greyscale (6 year old male, Case 2), demonstrating proliferative synovitis (*) and effusion (e). **(B)** Right wrist, 4^th^ compartment, longitudinal view, color Doppler (6 year old male, Case 2), demonstrating intense 3+ Doppler signal (D) in proliferative synovium.

**Figure 7:**
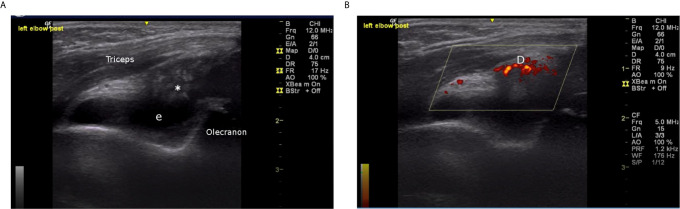
**(A)** Left elbow, posterior longitudinal view, greyscale (10 year old male, Case 6), demonstrating dense proliferative synovitis (*) in olecranon fossa with effusion (e). **(B)** Left elbow, posterior longitudinal view, color Doppler (10 year old male, Case 6), demonstrating intense Doppler signal (D) in olecranon fossa.

The small size of this series limited statistical power. Participants were recruited from a single clinical service, based at two referral centres in Cape Town, South Africa. Health systems in this region differ considerably from the rest of the continent, potentially limiting the generalisability of these findings to other settings and populations. The study was limited by a retrospective design and the availability of data in existing case files, laboratory records, and imaging reports. Sonographic data were only available for approximately two thirds of participants. Number of active joints was used as an objective surrogate marker of disease activity, as physician- and parent/patient-rated severity scores were not available for every follow-up encounter. It was not possible to assess a possible correlation between arthropathy disease activity and HIV viral load, as the timeframes over which viral load data and serial rheumatological assessment data were available differed in 7/11 cases. This study was further limited by a high rate of attrition. The study describes a series of highly vulnerable children living with a chronic health condition. Many of these children endure adverse social conditions, including poverty, parental and sibling mortality, household substance abuse, and inconsistent primary caregiver relationships, which may have contributed to loss to follow-up. Additionally, these chronically ill children have a significant risk of death, which may have contributed to attrition. In the comparable setting of paediatric ART clinics, several reviews have described high attrition rates (101–105).

A large prospective study would be beneficial to better define the characteristics, diagnosis, and optimal management of HIV arthropathy in children. Translational research is needed to investigate disease pathogenesis and identify biomarkers that could aid diagnosis in under-resourced environments without access to specialist assessment.

This study contributes to the small body of evidence on paediatric HIV arthropathy. Cases predominantly presented in older boys with advanced immunosuppression. The clinical presentation was variable, with both oligoarthritis and polyarthritis being common. Response to therapy was slower than has generally been described in adults, although fair outcomes were obtained with immunomodulating and antiretroviral therapy. Prospective and translational research is required to improve our understanding of this understudied disease.

## Data Availability Statement

The original contributions presented in the study are included in the article/**Supplementary Material**. Further inquiries can be directed to the corresponding author.

## Ethics Statement

The studies involving human participants were reviewed and approved by Human Research Ethics Committee Faculty of Health Sciences University of Cape Town. Written informed consent from the participants’ legal guardian/next of kin was not required to participate in this study in accordance with the national legislation and the institutional requirements.

## Author Contributions

All authors (MH, NB, and CS) contributed to the conception and design of this study. The data were collected by MH and CS. Data analysis was performed by MH, with input from CS. All authors interpreted the data. MH and CS drafted this work. All authors contributed to the article and approved the submitted version.

## Conflict of Interest

The authors declare that the research was conducted in the absence of any commercial or financial relationships that could be construed as a potential conflict of interest.

The reviewer AM declared a past co-authorship with one of the authors CS to the handling Editor.

## Abbreviations

ALC, Absolute lymphocyte count; ANC, Absolute neutrophil count; ART, Antiretroviral therapy; CLQ, Chloroquine; CMV, Cytomegalovirus; CRP, C-reactive protein; DIPJ, Distal interphalangeal joint; ESR, Erythrocyte sedimentation rate; Hb, Haemoglobin; HIV, Human immunodeficiency virus; HLA, Human leucocyte antigen; HREC, Human Research Ethics Committee; HSV, Herpes simplex virus; IPJ, Interphalangeal joint; IQR, Interquartile range; IRIS, Immune reconstitution inflammatory syndrome; LAD, Lymphadenopathy; MCPJ, Metacarpophalangeal joint; MTX, Methotrexate; NSAID, Nonsteroidal anti-inflammatory drug; PIPJ, Proximal interphalangeal joint; RA, Rheumatoid arthritis; SLE, Systemic lupus erythematosus; TB, Tuberculosis; uL, Microlitre; UWFA, Underweight-for-age; VPS, Ventriculoperitoneal shunt; VZV, Varicella zoster virus; WCC, White cell count; WFA, Weight-for-age.

## References

[1] Patel N Patel N Espinoza LR. HIV Infection and Rheumatic Diseases: The Changing Spectrum of Clinical Enigma. Rheum Dis Clin North Am (2009) 35(1):139–61. 10.1016/j.rdc.2009.03.007 19481002

[2] Fox C Walker-Bone K. Evolving Spectrum of HIV-Associated Rheumatic Syndromes. Best Pr Res Clin Rheumatol (2015) 29(2):244–58. 10.1016/j.berh.2015.04.019 PMC4759923 26362742

[3] Maganti RM Reveille JD Williams FM. The Changing Spectrum of Rheumatic Disease in HIV Infection. Nat Clin Pract (2008) 4(8):428–38. 10.1038/ncprheum0836 18577999

[4] Lau C-S Li P. The Effects of AIDS on the Prevalence of Rheumatic Diseases. Nat Epidemiol. (2016) 13(8). 10.1038/nrrheum.2016.196 27881863

[5] Buskila D Gladman DD Langevitz P Bookman AAM Fanning M Salit IE. Rheumatologic Manifestations of Infection With the Human Immunodeficiency Virus (HIV). Clin Exp Rheumatol (1990) 8(6):567–73. 2289325

[6] Fernandez SM Cardenal A Balsa A Quiralte J del Arco A Peña JM. Rheumatic Manifestations in 556 Patients With Human Immunodeficiency Virus Infection. Semin Arthritis Rheum (1991) 21(1):30–9. 10.1016/0049-0172(91)90054-4 1948099

[7] Medina F Pérez-Saleme L Moreno J. Rheumatic Manifestations of Human Immunodeficiency Virus Infection. Infect Dis Clin North Am (2006) 20(4):891–912. 10.1016/j.idc.2006.09.002 17118295

[8] Berman A Espinoza L Diaz J Aguilar J Rolando T Vasey F. Rheumatic Manifestations of Human Immunodeficiency Virus Infection. Am J Med (1988) 85(1):59–64. 10.1016/0002-9343(88)90503-7 3260453

[9] United Nations Joint Programme on HIV/AIDS. UNAIDS Data 2020. Geneva, Switzerland: UNAIDS (2020) p. 80–1.

[10] Hall K Sambu W. Demography of South Africa’s children. Cape Town, South Africa: Children’s Institute (2018).

[11] United Nations Joint Programme on HIV/AIDS. UNAIDS Data 2019. Geneva, Switzerland: UNAIDS (2019) p. 62–3.

[12] Ndirangu J Newell M-L Tanser F Herbst AJ Bland R. Decline in Early Life Mortality in a High HIV Prevalence Rural Area of South Africa: Evidence of HIV Prevention or Treatment Impact? AIDS (2014) 24(4):593–602. 10.1097/QAD.0b013e328335cff5 PMC4239477 20071975

[13] Zanoni BC Phungula T Zanoni HM France H Feeney ME. Risk Factors Associated With Increased Mortality Among HIV-Infected Children Initiating Antiretroviral Therapy in South Africa. PloS One (2011) 6(7):4–9. 10.1371/journal.pone.0022706 PMC3146475 21829487

[14] Violari A Cotton MF Gibb DM Babiker AG Steyn J Madhi S. Early Antiretroviral Therapy and Mortality Among HIV-Infected Infants. N Engl J Med (2008) 359(21):2233–44. 10.1056/NEJMoa0800971 PMC2950021 19020325

[15] Sekigawa I Ogasawara H Kaneko H Hishikawa T Hashimoto H. Retroviruses and Autoimmunity. Intern Med (2001) 40(2):80–6. 10.2169/internalmedicine.40.80 11300167

[16] Eggena MP Barugahare B Jones N Okello M Mutalya S Kityo C. Depletion of Regulatory T Cells in HIV Infection Is Associated With Immune Activation. J Immunol (2005) 174:4407–14. 10.4049/jimmunol.174.7.4407 15778406

[17] Hong YHX Luo JXJ. Autoimmunity and Dysmetabolism of Human Acquired Immunodeficiency Syndrome. Immunol Res (2016) 64(3):641–52. 10.1007/s12026-015-8767-5 26676359

[18] Weyand C Goronzy J. HIV Infection and Rheumatic Diseases: Autoimmune Mechanisms in Immunodeficient Hosts. Z Rheumatol (1992) 51(2):55–64. 10.1136/ard.51.2.253 1535472

[19] Zandman-Goddard G Shoenfeld Y. HIV and Autoimmunity. Autoimmun Rev (2002) 1(6):329–37. 10.1016/S1568-9972(02)00086-1 12848988

[20] Li Z Nardi MA Karpatkin S. Role of Molecular Mimicry to HIV-1 Peptides in HIV-1–Related Immunologic Thrombocytopenia. Blood (2005) 106(2):572–6. 10.1182/blood-2005-01-0243 PMC1895171 15774614

[21] Gleason B Mirembe G Namuyonga J Okello E Lwabi P Lubega I. Prevalence of Latent Rheumatic Heart Disease Among HIV-Infected Children in Kampala, Uganda. J Acquir Immune Defic Syndr (2016) 71(2):196–9. 10.1097/QAI.0000000000000827 PMC4712089 26413847

[22] Hovis IW Namuyonga J Kisitu GP Ndagire E Okello E Longenecker CT. Decreased Prevalence of Rheumatic Heart Disease Confirmed Among HIV Positive Youth. Pediatr Infect Dis J (2020) 38(4):406–9. 10.1097/INF.0000000000002161 PMC6355385 30882733

[23] Adizie T Moots R Hodkinson B French N Adebajo A. Inflammatory Arthritis in HIV Positive Patients: A Practical Guide. BMC Infect Dis (2016) 16(100). 10.1186/s12879-016-1389-2 PMC4774153 26932524

[24] Tikly M Njobvu P McGill P. Spondyloarthritis in Sub-Saharan Africa. Curr Rheumatol Rep (2014) 16(421). 10.1007/s11926-014-0421-z 24744085

[25] Njobvu P McGill P Kerr H Jellis J Pobee J. Spondyloarthropathy and Human Immunodeficiency Virus Infection in Zambia. J Rheumatol (1998) 25(8):1553–9. 9712100

[26] Nguyen B Reveille JD. Rheumatic Manifestations Associated With HIV in the Highly Active Antiretroviral Therapy Era. Curr Opin Rheumatol (2009) 21(4):404–10. 10.1097/BOR.0b013e32832c9d04 19444116

[27] Díaz-Peña R Ouédraogo D-D Vazquez AL Sawadogo S López-Larrea C. Ankylosing Spondylitis in Three Sub-Saharan Populations: HLA-B27 and HLA-B14 Contribution. Tissue Antigens (2012) 80:14–6. 10.1111/j.1399-0039.2012.01879.x 22536779

[28] Tshabalala M Mellet J Pepper MS. Human Leukocyte Antigen Diversity: A Southern African Perspective. J Immunol Res (2015) 746151. 10.1155/2015/746151 26347896 PMC4549606

[29] Njobvu P Gill P. Psoriatic Arthritis and Human Immunodeficiency Virus Infection in Zambia. J Rheumatol (2000) 27(7):1699–702. 10914854

[30] Bileckot R Mouaya A Makuwa M. Prevalence and Clinical Presentations of Arthritis in HIV-Positive Patients Seen at a Rheumatology Department in Congo-Brazzaville. Rev Rhum Engl Ed (1998) 65(10):549–54. 9809357

[31] Stein C Davis P. Arthritis Associated With HIV Infection in Zimbabwe. J Rheumatol (1996) 23(3):506–11. 8832993

[32] Bijlsma J Derksen R Huber-Bruning O Borleffs J. Does AIDS “Cure” Rheumatoid Arthritis? Ann Rheum Dis (1988) 47(4):350–1. 10.1136/ard.47.4.350-b PMC1003522 3365035

[33] Yao Q Frank M Glynn M Altman RD Altman RD. Rheumatic Manifestations in HIV-1 Infected Inpatients and Literature Review. Clin Exp Rheumatol (2008) 26:799–806. 19032811

[34] Lordache L Launay O Bouchaud O Jeantils V Goujard C Boue F. Autoimmune Diseases in HIV-Infected Patients: 52 Cases and Literature Review. Autoimmun Rev2 (2014) 13(8):850–7. 10.1016/j.autrev.2014.04.005 24747058

[35] Zhang X Li H Li T Zhang F Han Y. Distinctive Rheumatic Manifestations in 98 Patients With Human Immunodeficiency Virus Infection in China. J Rheumatol (2007) 34(8):1760–4. 17659750

[36] Ouédraogo D Ntsiba H Tiendrébéogo ZJ Tiéno H Bokossa L Kaboré F. Clinical Spectrum of Rheumatologic Diseases in a Department of Rheumatology in Ouagadougou (Burkina Faso). Clin Rheumatol (2014) 33(3):385–9. 10.1007/s10067-013-2455-4 24716217

[37] Marquez J Restrepo CS Candia L Berman A Espinoza LR. Human Immunodeficiency Virus-Associated Rheumatic Disorders in the HAART Era. J Rheumatol (2004) 31(4):741–6. 15088301

[38] Calabrese L Kirchner E Shrestha R. Rheumatic Complications of Human Immunodeficiency Virus Infection in the Era of Highly Active Antiretroviral Therapy: Emergence of a New Syndrome of Immune Reconstitution and Changing Patterns of Disease. Eur PMC (2005) 35(3):166–74. 10.1016/j.semarthrit.2005.03.007 16325657

[39] Azeroual A Harmouche H Benjilali L Mezalek Z Adnaoui M Aouni M. Rheumatoid Arthritis Associated to HIV Infection. Eur J Intern Med (2008) 19(6):e34–5. 10.1016/j.ejim.2007.09.020 18848165

[40] Mody G Parke F Reveille J. Articular Manifestations of Human Immunodeficiency Virus Infection. Best Pr Res Clin Rheumatol (2003) 17(2):265–87. 10.1016/S1521-6942(03)00003-2 12787525

[41] Reveille JD Conant MA Duvic M. Human Immunodeficiency Virus-Associated Psoriasis, Psoriatic Arthritis, and Reiter’s Syndrome: A Disease Continuum? 1Arthritis Rheum (1990) 33(10):1574–8. 10.1002/art.1780331016 2222538

[42] Mody G Patel N. Articular syndromes in association with HIV infection. CME (2011) 29(8):320–1. 10.1007/978-0-387-79061-9_5137

[43] Berman A Reboredo G Spindler A Lasala M Lopez H Espinoza L. Rheumatic Manifestations in Populations at Risk for HIV Infection: The Added Effect of HIV. J Rheumatol (1991) 18(10):1564–7. 1765982

[44] Ekwom PE Oyoo GO Amayo EO Muriithi IM. Prevalence and Characteristics of Articular Manifestations in Human Immunodeficiency Virus Infection. East Afr Med J (2010) 87(10):408–14. 23057274

[45] Medina-Rodriguez F Guzman C Jara L Hermida C Alboukrek D Cervera H. Rheumatic Manifestations in Human Immunodeficiency Virus Positive and Negative Individuals: A Study of Two Populations With Similar Risk Factors. J Rheumatol (1993) 20(11):1880–4. 8308773

[46] Lawson E Walker-Bone K. The Changing Spectrum of Rheumatic Disease in HIV Infection. Br Med Bull (2012) 103:203–21. 10.1093/bmb/lds022 22879627

[47] Berman A Cahn P Perez H Spindler A Lucero E Paz S. Human Immunodeficiency Virus Infection Associated Arthritis: Clinical Characteristics. J Rheumatol (1999) 26(5):1158–62. 10332983

[48] Blanche P Taelman H Saraux A Bogaerts J Clerinx J Batungwanayo J. Acute Arthritis and Human Immunodeficiency Virus Infection in Rwanda. Eur PMC (1993) 20(12):2123–7. 8014942

[49] Rynes R Goldenberg D DiGiacomo R Olson R Hussain M Veazey J. Acquired Immunodeficiency Syndrome-Associated Arthritis. Am J Med (1988) 84(5):810–6. 10.1016/0002-9343(88)90057-5 3364441

[50] Chinniah K Mody GM Bhimma R Adhikari M. Arthritis in Association With Human Immunodeficiency Virus Infection in Black African Children: Causal or Coincidental? Rheumatology (2005) 44:915–20. 10.1093/rheumatology/keh636 15827039

[51] Espinoza L Aguilar J Espinoza C Berman A Gutierrez F Vasey F. HIV Associated Arthropathy: HIV Antigen Demonstration in the Synovial Membrane. J Rheumatol (1990) 17(9):1195–201. 2290161

[52] Hughes R Macatonia S Rowe I Keat A Knight S. The Detection of Human Immunodeficiency Virus DNA in Dendritic Cells From the Joints of Patients With Aseptic Arthritis. Br J Rheumatol (1990) 29:166–70. 10.1093/rheumatology/29.3.166 2141535

[53] Hochberg M Fox R Nelson K Saah A. HIV Infection Is Not Associated With Reiter’s Syndrome: Data From the John Hopkins Multicenter AIDS Cohort Study. AIDS (1990) 4(11):1149–51. 10.1097/00002030-199011000-00016 2282189

[54] Romanelli F Smith K Hoven A. Chloroquine and Hydroxychloroquine as Inhibitors of Human Immunodeficiency Virus (HIV-1) Activity. Curr Pharm Des (2004) 10(21):2643–8. 10.2174/1381612043383791 15320751

[55] Savarino A Boelaert JR Cassone A Majori G Cauda R. Effects of chloroquine on viral infections: An old drug against today’s diseases? Lancet (2003) 3(November):722–7. 10.1016/S1473-3099(03)00806-5 PMC7128816 14592603

[56] Devaux CA Rolain J Colson P Raoult D. New Insights on the Antiviral Effects of Chloroquine Against Coronavirus: What to Expect for COVID-19? Int J Antimicrob Agents. (2020) 55. 10.1016/j.ijantimicag.2020.105938 PMC7118659 32171740

[57] Zaborowski AG Parbhoo D Chinniah K Visser L. Uveitis in Children With Human Immunodeficiency Virus–Associated Arthritis. J Am Assoc Pediatr Ophthalmol Strabismus (2008) 12(6):608–10. 10.1016/j.jaapos.2008.04.011 18757220

[58] Lakshmanan P Shah I. Human Immunodeficiency Virus Polyarthropathy. J Fam Med Prim Care (2016) 5(3):725–6. 10.4103/2249-4863.197289 PMC5290797 28217620

[59] Zaki SA Tilak L Shanbag P. Arthritis as the Presenting Feature of HIV Infection in a Child. J Infect (2009) April):391–2. 10.1016/j.jinf.2009.03.002 19359043

[60] Chakladar D Mondal RK Sabui TK Bhowmik S Biswas T. Musculoskeletal Manifestations in Pediatric Patients Infected With Human Immunodeficiency Virus: Developing Country Perspective. Eur J Rheumatol (2019) 6(1):7–11. 10.5152/eurjrheum.2018.18045 30489252 PMC6459328

[61] Suri D Sharma A Bhattad S Rawat A Arora S Minz RW. Arthritis in Childhood Human Immunodeficiency Virus Infection Predominantly Associated With Human Leukocyte Antigen B27. Int J Rheum Dis (2016) 19(10):1018–23. 10.1111/1756-185X.12947 27456089

[62] Chipeta J Njobvu P Wa-Somwe S Chintu C McGill PE Bucala R. Clinical Patterns of Juvenile Idiopathic Arthritis in Zambia. Pediatr Rheumatol (2013) 11(33). 10.1186/1546-0096-11-33 PMC3848636 24034206

[63] Mahtab S Scott C Chris Machemedze T Joubert S Asafu-Agyei NA Myer L Zar HJ. Musculoskeletal conditions among perinatally HIV-infected adolescents in the Cape Town Adolescent Antiretroviral cohort. In: Proc 25th Eur Paediatric Rheumatol Congress (PReS 2018) Lisbon Portugal (2018), 16:50–1. 10.1186/s12969-018-0265-6

[64] Schuval S Bonagura V Ilowite N. Rheumatologic Manifestations of Pediatric Human Immunodeficiency Virus Infection. J Rheumatol (1993) 20(9):1578–82. 8164219

[65] Martínez-Rojano H Hernández EJ Guevara GL Gorbea-Robles M del C. Rheumatologic Manifestations of Pediatric HIV Infection. AIDS Patient Care STDS (2016) 15(10):519–26. 10.1089/108729101753205685 11689139

[66] Johnson L Dorrington R Moolla H. Progress Towards the 2020 Targets for HIV Diagnosis and Antiretroviral Treatment in South Africa. S Afr J HIV Med (2017) 18(1):a694. 10.4102/sajhivmed.v18i1.694 PMC5843157 29568630

[67] Aimes S. Management of Inflammatory Arthritis in Patients With Comorbid HIV Infection [Internet](2016). Available at: https://www.rheumatologyadvisor.com/home/topics/rheumatoid-arthritis/management-of-inflammatory-arthritis-in-patients-with-comorbid-hiv-infection/ (Accessed 2021 Jan 25).

[68] Ferrand RA Luethy R Bwakura F Mujuru H Miller RF Corbett EL. HIV Infection Presenting in Older Children and Adolescents: A Case Series From Harare, Zimbabwe. Clin Infect Dis (2007) 44(6):874–8. 10.1086/511878 17304463

[69] Kranzer K Bradley J Musaazi J Nyathi M Gunguwo H Ndebele W. Loss to follow-up among children and adolescents growing up with HIV infection: Age really matters. J Int AIDS Soc (2017) 20(1). 10.7448/IAS.20.1.21737 PMC5577636 28715158

[70] Birhan TY Gezie LD Teshome DF Sisay MM. Predictors of CD4 Count Changes Over Time Among Children Who Initiated Highly Active Antiretroviral Therapy in Ethiopia. Trop Med Health (2020) 48(1). 10.1186/s41182-020-00224-9 PMC7243309 32476985

[71] Scully EP. Sex Differences in HIV Infection. Curr HIV/AIDS Rep (2018) 15(2):136–46. 10.1007/s11904-018-0383-2 PMC5882769 29504062

[72] Farzadegan H Hoover D Astemborski J Lyles C Vlahov D. Sex Differences in HIV-1 Viral Load and Progression to AIDS. Lancet (1998) 352:1510–3. 10.1016/S0140-6736(98)02372-1 9820299

[73] Evans JS Nims T Cooley J Bradley W Jagodzinski L Zhou S. Serum Levels of Virus Burden in Early-Stage Human Immunodeficiency Virus Type 1 Disease in Women. J Infect Dis (1997) 175(4):795–800. 10.1086/513973 9086132

[74] Touloumi G Pantazis N Babiker A Walker S Katsarou O Karafoulidou A. Differences in HIV RNA Levels Before the Initiation of Antiretroviral Therapy Among 1864 Individuals With Known HIV-1 Seroconversion Dates. AIDS2 (2004) 18(12):1697–705. 10.1097/01.aids.0000131395.14339.f5 15280781

[75] Vieira VA Zuidewind P Muenchhoff M Roider J Millar J Clapson M. Strong Sex Bias in Elite Control of Paediatric HIV Infection. Aids (2019) 33(1):67–75. 10.1097/QAD.0000000000002043 30325765 PMC6750143

[76] Crowell TA Gebo KA Blankson JN Korthuis PT Yehia BR Rutstein RM. Hospitalization Rates and Reasons Among HIV Elite Controllers and Persons With Medically Controlled HIV Infection. J Infect Dis (2015) 211(11):1692–702. 10.1093/infdis/jiu809 PMC4447832 25512624

[77] Madec Y Boufassa F Porter K Meyer L. Spontaneous Control of Viral Load and CD4 Cell Count Progression Among HIV-1 Seroconverters. AIDS (2005) 19(17):2001–7. 10.1097/01.aids.0000194134.28135.cd 16260907

[78] de Azevedo SSD Caetano DG Côrtes FH Teixeira SLM Santos Silva K Hoagland B. Highly divergent patterns of genetic diversity and evolution in proviral quasispecies from HIV controllers. Retrovirology (2017) 14(29). 10.1186/s12977-017-0354-5 PMC5414336 28464889

[79] Raghavan A Rimmelin DE Fitch KV Zanni MV. Sex Differences in Select non-Communicable HIV-Associated Comorbidities: Exploring the Role of Systemic Immune Activation/Inflammation. Curr HIV/AIDS Rep (2017) 14(6):220–8. 10.1007/s11904-017-0366-8 PMC6007989 29080122

[80] Meier A Chang JJ Chan ES Pollard RB Sidhu HK Kulkarni S. Sex Differences in the Toll-Like Receptor-Mediated Response of Plasmacytoid Dendritic Cells to HIV-1. Nat Med (2009) 15(8):955–9. 10.1038/nm.2004 PMC2821111 19597505

[81] Chang JJ Woods M Lindsay RJ Doyle EH Griesbeck M Chan ES. Higher Expression of Several Interferon-Stimulated Genes in HIV-1-Infected Females After Adjusting for the Level of Viral Replication. J Infect Dis (2013) 208(5):830–8. 10.1093/infdis/jit262 PMC3733517 23757341

[82] Newell ML Coovadia H Cortina-Borja M Rollins N Gaillard P Dabis F. Mortality of Infected and Uninfected Infants Born to HIV-Infected Mothers in Africa: A Pooled Analysis. Lancet (2004) 364(9441):1236–43. 10.1016/S0140-6736(04)17140-7 15464184

[83] Little K Thorne C Luo C Bunders M Ngongo N McDermott P. Disease Progression in Children With Vertically-Acquired HIV Infection in Sub-Saharan Africa: Reviewing the Need for HIV Treatment. Curr HIV Res (2007) 5(2):139–53. 10.2174/157016207780077002 17346131

[84] Spira R Lepage P Msellati P Van De Perre P Leroy V Simonon A. Natural history of human immunodeficiency virus type 1 infection in children: A five-year prospective study in Rwanda. Pediatrics (1999) 104(5). 10.1542/peds.104.5.e56 10545582

[85] Jean S Pape J Verdier R Reed G Hutto C Johnson W. The Natural History of Human Immunodeficiency Virus 1 Infection in Haitian Infants. Pediatr Infect Dis J (1999) 18(1):58–63. 10.1097/00006454-199901000-00014 9951982

[86] Collaborative Group on AIDS Incubation and HIV Survival including the CASCADE EU Concerted Action. Time from HIV-1 seroconversion to AIDS and death before widespread use of highly-active antiretroviral therapy: A collaborative re-analysis. Lancet (2000) 355(9210):1131–7. 10.1016/S0140-6736(00)02061-4 10791375

[87] Paul ME Mao C Charurat M Serchuck L Foca M Hayani K. Predictors of Immunologic Long-Term Nonprogression in HIV-Infected Children: Implications for Initiating Therapy. J Allergy Clin Immunol (2005) 115(4):848–55. 10.1016/j.jaci.2004.11.054 15806009

[88] Goulder PJ Lewin SR Leitman EM. Paediatric HIV Infection: The Potential for Cure. Nat Rev Immunol (2016) 16(4):259–71. 10.1038/nri.2016.19 PMC5694689 26972723

[89] Kumar P. Long Term non-Progressor HIV Infection. Indian J Med Res (2013) 138(3):291–3. PMC3818590 24135172

[90] Limou S Le Clerc S Coulonges C Carpentier W Dina C Delaneau O. Genomewide Association Study of an AIDS-Nonprogression Cohort Emphasizes the Role Played by HLA Genes (ANRS Genomewide Association Study 02). J Infect Dis (2009) 199(3):419–26. 10.1086/596067 19115949

[91] den Uyl D van der Horst-Bruinsma I van Agtmael M. Progression of HIV to AIDS: A Protective Role for HLA-B27? AIDS Rev (2004) 6:89–96. 15332431

[92] López-Larrea C Njobvu PD González S Blanco-Gelaz MA Martínez-Borra J López-Vázquez A. The HLA-B*5703 Allele Confers Susceptibility to the Development of Spondylarthropathies in Zambian Human Immunodeficiency Virus-Infected Patients With Slow Progression to Acquired Immunodeficiency Syndrome. Arthritis Rheum (2005) 52(1):275–9. 10.1002/art.20722 15641063

[93] Chen H Hayashi G Lai OY Dilthey A Kuebler PJ Wong TV. Psoriasis patients Are enriched for genetic variants that protect against HIV-1 disease. PloS Genet (2012) 8(2). 10.1371/journal.pgen.1002514 PMC3343879 22577363

[94] Neumann-Haefelin C. HLA-B27-Mediated Protection in HIV and Hepatitis C Virus Infection and Pathogenesis in Spondyloarthritis: Two Sides of the Same Coin? Curr Opin Rheumatol (2013) 25(4):426–33. 10.1097/BOR.0b013e328362018f 23656712

[95] Plate A Boyle B. Musculoskeletal Manifestations of HIV Infection. AIDS Read (2003) 13(2):69–76. 12645490

[96] Mahajan A Tandon V Verma S. Rheumatological Manifestations in HIV Infection. J Indian Acad Clin Med (2006) 7(2):136–44.

[97] Restrepo CS Lemos DF Gordillo H Odero R Varghese T Tiemann W. Imaging Findings in Musculoskeletal Complications of AIDS. Radiographics (2004) 24(4):1029–49. 10.1148/rg.244035151 15256627

[98] Rosenberg Z Norman A Solomon G. Arthritis Associated With HIV Infection: Radiographic Manifestations. Radiology (1989) 173(1):171–6. 10.1148/radiology.173.1.2781004 2781004

[99] Allroggen A Frese A Rahmann A Gaubitz M Husstedt I Evers S. HIV Associated Arthritis: Case Report and Review of the Literature. Eur J Med Res (2005) 10(7):305–8. 16055402

[100] Reveille J Williams M. Rheumatologic Complications of HIV Infection. Best Pract Res Clin Rheumatol (2006) 20(6):1159–79. 10.1016/j.berh.2006.08.015 17127202

[101] KIDS-ART-LINC Collaboration. Low Risk of Death, But Substantial Program Attrition in Pediatric HIV Treatment Cohorts in Sub-Saharan Africa. J Acquir Immune Defic Syndr (2008) 49(5):523–31. 10.1097/QAI.0b013e31818aadce 18989227

[102] Carlucci JG Liu Y Clouse K Vermund SH. Attrition of HIV-Positive Children From HIV Services in Low and Middle-Income Countries. AIDS (2019) 33(15):2375–86. 10.1097/QAD.0000000000002366 PMC6905128 31764102

[103] Abuogi LL Smith C Mcfarland EJ. Retention of HIV-Infected Children in the First 12 Months of Anti-Retroviral Therapy and Predictors of Attrition in Resource Limited Settings : A Systematic Review. PloS One (2016) 11(6):e0156506. 10.1371/journal.pone.0156506 27280404 PMC4900559

[104] Lamb MR Fayorsey R Nuwagaba-biribonwoha H Viola V Mutabazi V Alwar T. High Attrition Before and After ART Initiation Among Youth (15–24 Years of Age) Enrolled in HIV Care. AIDS (2014) 28(4):559–68. 10.1097/QAD.0000000000000054 PMC4517438 24076661

[105] Fox MP Shearer K Maskew M Meyer-rath G Clouse K Sanne I. Attrition Through Multiple Stages of Pre-Treatment and ART HIV Care in South Africa. PloS One (2014) 9(10):e110252. 10.1371/journal.pone.0110252 25330087 PMC4203772

